# A Comprehensive Review of Radiomic Methodologies for Assessing Tumor Response to Immunotherapy and Combination Therapies

**DOI:** 10.2174/0115734056404322251203083041

**Published:** 2026-03-09

**Authors:** Haijun Tian, Yawei Ma, Li Zhu, Yongle Yu, Youfu Ma, Xialing Huang, Xu Liu

**Affiliations:** 1 Guangxi Key Laboratory of Special Biomedicine; School of Medicine, Guangxi University, Nanning, 530004, Guangxi Zhuang Autonomous Region, China; 2 Department of Radiology, The First Affiliated Hospital of Guangxi Medical University, Nanning 530021, Guangxi Zhuang Autonomous Region, China

**Keywords:** Radiomics, Immunotherapy, CT, PET, MRI, RECIST

## Abstract

Recent advances in the genetic and molecular characterization of cancer have significantly enhanced the development of immunotherapies, which bolster the host immune system’s ability to recognize and target malignant cells, thereby promoting immune responses and inhibiting tumor progression and metastasis. Despite these promising developments, not all patients benefit from immunotherapy, underscoring the urgent need for reliable biomarkers to facilitate early, predictive identification of responders and non-responders. Conventional methods for evaluating treatment response, such as the World Health Organization (WHO) criteria and the Response Evaluation Criteria in Solid Tumors (RECIST), which were primarily designed for cytotoxic chemotherapy, exhibit notable limitations. These criteria may fail to detect hyperprogressive disease in a timely manner, thereby hindering optimal treatment decision-making. Recent technological advancements in imaging modalities, including CT, PET, MRI, and radiomics, have provided critical functional and metabolic insights that enhance the evaluation and prediction of responses to immunotherapy. This review consolidates the latest findings from publicly available research and WHO reports, with a particular focus on high-mortality cancer types, and examines recent developments in non-invasive methodologies for predicting and assessing the therapeutic efficacy of immunotherapy and its combination therapies.

## 1 INTRODUCTION

Immunotherapy, an emerging therapeutic modality, holds considerable promise in enhancing both the survival and quality of life for cancer patients, unlike traditional therapies, which directly target and eliminate tumor cells. Immunotherapy functions by stimulating the immune system to autonomously recognize and attack cancerous cells, thereby eliciting sustained therapeutic effects and the formation of immune memory. Furthermore, immunotherapy can be combined effectively with other treatment modalities, such as chemotherapy and radiotherapy, thereby broadening the therapeutic options.

A major clinical challenge in the field is predicting which patients are likely to benefit from immunotherapy. Identifying individuals who exhibit treatment resistance is critical, as it conserves resources, minimizes unnecessary interventions, and reduces the risk of adverse effects. Immunotherapy often induces distinctive response patterns-such as initial tumor enlargement followed by subsequent shrinkage or the emergence of new lesions-that are not adequately captured by conventional response criteria [1]. Given that RECIST 1.1 fails to account for such changes in metabolic and functional status, new evaluation standards, including irRC, irRECIST, and iRECIST, have been developed to more effectively differentiate between responders and non-responders (Table **1**) [1-11]. Imaging techniques such as Computed Tomography (CT), Positron Emission Tomography (PET), Magnetic Resonance Imaging (MRI), and radiomics are becoming increasingly indispensable for assessing the response to immunotherapy. These methods provide valuable insights into the metabolic and functional characteristics of tumors, addressing the limitations of traditional evaluation criteria. For instance, PET/CT can distinguish between residual tumors and surrounding tissue, thereby enabling more accurate predictions of treatment response in patients with stable disease than RECIST. Radiomics, on the other hand, involves the extraction of advanced quantitative features from medical images, enabling a non-invasive assessment of the tumor microenvironment, spatial heterogeneity, and disease progression [12].

This review synthesizes the most recent data from the World Health Organization (WHO) and focuses on high-mortality cancer types. It highlights recent advancements in non-invasive methodologies for predicting and evaluating tumor responses to immunotherapy and combination therapies.

## 2 METHODS

Relevant literature was identified through a comprehensive search of PubMed, Web of Science, and Embase databases up to June 2024. The search strategy used keywords related to “radiomics,” “immunotherapy,” “combination therapy,” “solid tumors,” and specific tumor types (*e.g*., “hepatocellular carcinoma,” “lung cancer,” “colorectal cancer,” “melanoma”).

Studies were included if they met the following criteria: (1) published in English in peer-reviewed journals; (2) focused on radiomics methodologies for evaluating immunotherapy or combination treatment outcomes; and (3) reported original research data, retrospective or prospective analyses, or systematic reviews with sufficient methodological details. Conference abstracts, editorials, commentaries, and studies lacking methodological clarity were excluded.

After duplicates were removed, the author screened titles and abstracts, followed by full-text assessment according to predefined criteria. Key information on study design, cancer type, imaging modality, segmentation approach, feature extraction, algorithms used, validation strategies, and model performance was systematically extracted. Findings were then synthesized narratively to outline current methodological trends, compare insights across cancer types, and highlight existing limitations, emerging standards, and future directions for clinical application.

## 3 OVERVIEW OF PROGRESS IN RADIOMICS FOR SOLID TUMOR IMMUNOTHERAPY

Radiomics studies for solid tumor immunotherapy typically rely on three main imaging modalities, CT, MRI, and PET, each offering distinct advantages and complementary insights into tumor biology.

CT remains the cornerstone of oncologic imaging and the most accessible tool worldwide, favored for its fast acquisition, high spatial resolution, and well-established role in routine staging and treatment monitoring. CT-based radiomics is particularly effective for quantifying morphological heterogeneity, tumor borders, necrosis, and subtle textural patterns within primary and metastatic lesions. Multi-phase contrast-enhanced CT is widely used for liver, lung, and gastrointestinal tumors, enabling the extraction of dynamic vascular features that correlate with perfusion and angiogenesis, which are relevant for predicting immune response and treatment-induced changes.

MRI provides superior soft tissue contrast and diverse functional sequences, making it indispensable for tumors where anatomical detail and tissue characterization are critical. Beyond standard T1- and T2-weighted sequences, advanced techniques such as Diffusion-Weighted Imaging (DWI) can assess tumor cellularity, while Dynamic Contrast-Enhanced (DCE) MRI can evaluate microvascular permeability and tumor perfusion. These parameters have proven useful for identifying immunotherapy responders in liver cancer, brain tumors, and pelvic malignancies such as rectal or cervical cancer. Moreover, emerging studies combine multiparametric MRI with radiogenomic data to explore associations between imaging phenotypes and immune-related genomic profiles.

PET, most commonly performed with Fluorodeoxyglucose (FDG), adds a metabolic dimension that complements the anatomical details provided by CT and MRI. Metabolic parameters, including SUVmax, Metabolic Tumor Volume (MTV), and total lesion glycolysis (TLG), are widely recognized predictors of treatment response and have been incorporated into radiomic models for lung cancer, lymphoma, melanoma, and other solid tumors with high metabolic activity. PET/CT fusion imaging allows precise co-registration of metabolic and structural information, enhancing the accuracy of tumor delineation and feature extraction.

One of the primary objectives of radiomics in the immunotherapy era is to non-invasively characterize tumor biological heterogeneity and the tumor immune microenvironment, which are critical determinants of treatment response. Several core biomarkers have been extensively studied across different solid tumors. Programmed Death-Ligand 1 (PD-L1) expression remains the most established predictive biomarker for Immune Checkpoint Inhibitor (ICI) efficacy, yet its assessment by biopsy is limited by sampling bias and intratumoral heterogeneity. TIME is pivotal in tumor development, metastasis, and treatment response [13]. Numerous studies have confirmed the prognostic and predictive significance of immune cell infiltration in tumors. Microsatellite Instability (MSI) status, especially relevant for colorectal and gastric cancers, reflects defective DNA mismatch repair and is associated with higher neoantigen loads, leading to better immunotherapy outcomes [14]. Tumor Mutational Burden (TMB) quantifies the total number of somatic mutations and serves as a surrogate for the likelihood of neoantigen formation; radiogenomic strategies are increasingly exploring non-invasive TMB prediction using multimodal imaging features.

In addition to these well-known markers, Tumor-Infiltrating Lymphocytes (TILs) and composite immunoscore frameworks have gained attention as robust predictors of the host immune response. High TIL density often indicates an active anti-tumor immune environment and has been shown to correlate with better outcomes in melanoma, lung, and colorectal cancers. Radiomic models have been developed to estimate TIL density and spatial distribution from CT or MRI scans by analyzing peritumoral textures and vascular features. The immunoscore, which combines the density and localization of TILs within the tumor core and invasive margin, has been validated as a superior prognostic tool in colorectal cancer and is now being investigated in other solid tumors. By integrating radiomic features that reflect immune cell infiltration, researchers aim to provide a non-invasive surrogate for immunoscore estimation.

Lastly, metabolic markers derived from FDG-PET, such as maximum Standardized Uptake Value (SUVmax), Metabolic Tumor Volume (MTV), and Total Lesion Glycolysis (TLG), remain widely used quantitative parameters for assessing tumor metabolic activity and treatment response. These PET-based features have been incorporated into radiomic pipelines to enhance prognostic accuracy in lung cancer, lymphomas, and melanoma. Together, these biomarkers illustrate how radiomics can complement or potentially replace tissue-based assays, paving the way for individualized prediction of immunotherapy response.

Over the past decade, radiomics has undergone substantial evolution in the context of immunotherapy, shifting from exploratory analyses of handcrafted features to advanced, deep learning–driven, multimodal frameworks.

In the early phase, research primarily focused on extracting engineered texture, shape, and intensity features from CT or MRI to identify imaging biomarkers of immune response. These studies predominantly employed traditional machine learning classifiers, such as support vector Machines (SVMs), LASSO regression, and random forests, which, although interpretable, required extensive manual segmentation and were limited in their ability to capture complex imaging phenotypes.

Between 2018 and 2020, the field advanced toward more sophisticated modeling approaches, incorporating machine–learning–based predictive frameworks, immune-related response criteria, and integration with clinical and molecular biomarkers. This period marked a shift toward more comprehensive characterization of tumor–immune interactions.

Since 2021, radiomics research has increasingly embraced deep learning architectures that automatically learn hierarchical representations from raw imaging data, thereby reducing reliance on manual feature engineering. Convolutional Neural Network (CNN) variants, such as U-Net and 3D U-Net, are widely applied for tumor segmentation and volumetric feature extraction [15, 16]. Residual Networks (ResNets) and DenseNets frequently serve as backbone architectures in classification pipelines, whereas attention mechanisms and hybrid models enhance feature weighting and spatial interpretability [17]. More recently, transformer-based frameworks, such as Vision Transformers (ViTs) and Swin Transformers, have demonstrated considerable potential for capturing multi-scale spatial relationships in advanced segmentation and treatment response prediction [18]. In addition, Generative Adversarial Networks (GANs) have been employed for data augmentation and domain adaptation, particularly to address the challenges of small sample sizes and multi-center variability [19].

**Fig. (1):**
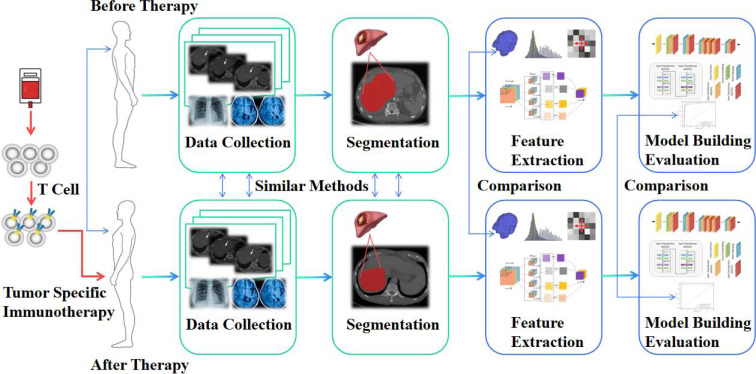
Conceptual workflow summarizing the typical radiomics pipeline for immunotherapy prediction.

Collectively, these methodological advances underpin a new generation of radiomics pipelines that are more automated, integrative, and clinically aligned, thereby meeting the demands of precision immuno-oncology. A conceptual workflow diagram (Fig. **1**) illustrates the standardized methodological framework, outlining key stages: data acquisition, tumor segmentation, feature extraction, model development, and performance evaluation. Systematic integration of these stages is essential for developing robust, generalizable radiomics models that can inform clinical decision-making in both immunotherapy and combination treatment contexts.

## 4 RADIOMICS FOR MAJOR SOLID TUMORS

### 4.1 Hepatocellular Carcinoma

According to the most recent data from the WHO, liver cancer is the fourth leading cause of cancer-related mortality worldwide [20]. Current treatment options include surgery, local ablation, transarterial chemoembolization, radiotherapy, and systemic therapy. Due to nonspecific early symptoms, many patients are diagnosed at advanced stages and miss the window for curative resection, resulting in poor prognosis and high recurrence rates. Recent studies show that tyrosine kinase inhibitors such as sorafenib can prolong survival in advanced HCC, and their combination with immunotherapy or local treatments is being actively explored to improve outcomes.

Several studies have confirmed the complementary value of ^18^F-FDG-PET/CT alongside conventional criteria such as RECIST and mRECIST for evaluating treatment response in HCC. PET/CT-derived metrics, especially the tumor-to-liver uptake ratio (TLR), can reflect tumor metabolic behavior more reliably than blood flow-based indicators alone. In lenvatinib-treated patients, TLR dynamics correlate with AFP changes and histological differentiation, suggesting potential as a surrogate marker of response [21]. By contrast, in Atezolizumab plus Bevacizumab (Atezo/Bev) cohorts, a higher pre-treatment TLR (≥2) has been linked to higher rates of early progression and poorer survival, underscoring how tumor biology and treatment history can shape FDG uptake patterns [22]. Additionally, Zhou *et al*. [23] showed that SUVmax in primary HCC tumors correlates with poor differentiation, high tumor burden, portal vein tumor thrombus, lymph node metastasis, and increased mortality risk, and is strongly linked to PD-L1 expression.

Gadolinium Ethoxybenzyl Diethylenetriamine Pentaacetic acid (Gd-EOB-DTPA) is a liver-specific MRI contrast agent, preferentially absorbed by hepatocytes *via* OATP1B3. In the Hepatobiliary Phase (HBP) of EOB-MRI, typical HCC generally exhibits lower intensity compared to the surrounding liver parenchyma [24]. Studies suggest that Gd-EOB-DTPA-enhanced MRI is a valuable method for identifying β-catenin mutations. Kudo M. indicated that Gd-EOB-DTPA-enhanced MRI could predict Wnt/β-catenin mutations and resistance to immune checkpoint inhibitors in HCC patients [25]. Similarly, Sasaki *et al*. [26] proposed that the HBP phase of Gd-EOB-DTPA-enhanced MRI could be useful in predicting the therapeutic efficacy of atezolizumab combined with bevacizumab in treating unresectable HCC. Aoki *et al*. [27] demonstrated that high enhancement of intrahepatic nodules on HBP Gd-EOB-DTPA-enhanced MRI serves as a poor prognostic marker in unresectable hepatocellular carcinoma treated with anti-PD-1/PD-L1 monotherapy. Sun *et al*. [28] employed Gd-EOB-DTPA-enhanced MRI to predict CD8 cell density and PD-L1 expression in HCC tumors. The results revealed that MRI features, including irregular tumor edges, low signal intensity in the HBP, and absence of cysts, may help identify HCC patients with an activated immune state and predict immunotherapy outcomes.

Recent innovations show that radiomics models can noninvasively characterize the Tumor Immune Microenvironment (TIME) and predict responses to immunotherapy or combination treatments in HCC. Preclinical studies show that MRI texture features from T1/T2-weighted sequences can distinguish NK cell, Sorafenib, and combination treatment outcomes, overcoming the limitations of size-based criteria such as iRECIST [29]. Similarly, CT-based RNETS models combine NETs gene signatures with texture features to estimate immune status and predict anti-PD-1 benefit, offering a practical alternative when molecular tests are limited [30]. Additionally, Gd-EOB-DTPA MRI and deep learning–radiomics pipelines predict PD-L1 status and Immunoscore, stratifying TIME into phenotypes linked to prognosis and immunotherapy response [31]. While these models show promise for improving risk stratification and personalized planning, challenges such as small cohorts, tumor heterogeneity, and unmeasured clinical variables (*e.g*., NASH, HBV infection, cirrhosis) underline the need for multicenter validation and further refinement. Future studies should combine radiomics with molecular and clinical data to enable broader translation in advanced HCC. (Table **2**) [30-33].

Beyond conventional HCC, rare primary liver tumors such as combined Hepatocellular-Cholangiocarcinoma (cHCC-CCA) and Intrahepatic Cholangiocarcinoma (ICC) have attracted increasing attention due to their diagnostic and therapeutic challenges. However, current radiomics research on these non-HCC liver tumors remains limited and mostly exploratory, partly due to their low incidence and diverse histopathological features. Preliminary studies have suggested that multiphasic CT and MRI radiomics may help differentiate cHCC-CCA from HCC and ICC and provide prognostic insights. However, robust validation in larger, multi-center cohorts is still lacking. Therefore, future research should prioritize standardized imaging protocols and multi-modal radiomic frameworks to extend the benefits of radiomics beyond HCC.

### 4.2 Lung Cancer

In 2022, global malignant tumor incidence reached 19,976,499 cases, an increase of 700,000 from 2020 [20]. Lung cancer, the most common type, accounted for 2,480,675 cases (12.4%) and the highest mortality rate [20]. The WHO classifies lung cancer into two primary histological types: Non-Small Cell Lung Cancer (NSCLC), which accounts for 80%-85% of cases, and Small Cell Lung Cancer (SCLC), which constitutes approximately 15%. While early-stage lung cancer may be treated surgically, advanced stages often require systemic therapies. In recent years, immune checkpoint inhibitors such as nivolumab, pembrolizumab, and atezolizumab have been approved for advanced NSCLC, significantly improving survival in selected patients [34].

Radiomics analyses have revealed that quantitative imaging features, including shape, size, and intensity, closely correlate with gene and protein expression in NSCLC. A multicenter study developed a Deep Learning Score (DLS) that demonstrated high discriminative performance in training, internal validation, and independent external cohorts (AUC 0.82–0.89; C-index 0.81–0.87). Early fusion of PET and CT features and focusing on tumor-segmented regions significantly enhanced model performance compared to single-modality or whole-lung approaches [35]. Da-Ano *et al*. [36] compared different network architectures (ResNet, DenseNet, EfficientNet) and fusion strategies, showing that early fusion and separate encoding of metabolic (PET) and anatomical (CT) information yielded better performance, highlighting the importance of leveraging complementary metabolic and anatomical features. In addition to multi-modal PET/CT approaches, a recent study developed a novel CT-only radiogenomics biomarker (LCI-RPV) to predict PD-L1 positivity, 3-month treatment response, and the risk of immunotherapy-related pneumonitis. This model integrates radiomics features from the tumor, peritumoral regions, and lung parenchyma, and demonstrated robust validation across heterogeneous multi-center datasets, indicating good generalizability. It may serve as a supplementary decision-making tool when tissue sampling is limited [37]. Seban *et al*. [38] and Kudure *et al*. [39] further confirmed that combining baseline FDG-PET/CT metabolic parameters with clinical and morphological features enhances treatment outcome prediction accuracy in NSCLC patients. (Table **3**) [36, 40-42].

SCLC typically shows high metabolic activity on FDG-PET/CT, with higher pre-treatment SUVmax values in extensive-stage tumors linked to poorer prognosis. In limited-stage SCLC, the 2-year survival rate is lower in the high SUVmax group than in the low SUVmax group [43]. Pre-treatment total lesion glycolysis and metabolic tumor volume also correlate with prognosis, with higher glycolysis linked to significantly shorter median OS, and larger metabolic tumor volume associated with shorter PFS [44]. In a multicenter study, high baseline TMTV, measured by pretreatment FDG PET/CT, predicted poor prognosis in ES-SCLC patients receiving first-line Chemoimmunotherapy (CIT). Combining TMTV with liver metastasis status further stratified patients into distinct risk groups, demonstrating the added value of this composite metabolic-imaging biomarker for guiding personalized treatment planning. Notably, tumor SUVmax alone did not correlate with survival, underscoring the superior prognostic relevance of TMTV and liver metastasis [45]. In parallel, Machine Learning (ML)-based radiomics models using pretreatment chest CT have shown promise for non-invasively predicting outcomes in ES-SCLC. Zhao *et al*. [41] developed the first large-scale multicenter ML-based radiomics nomogram, demonstrating that radiomics features combined with clinical indicators significantly improve prediction accuracy for PFS and OS compared to clinical data alone. The model achieved AUCs of 0.635–0.733 and effectively stratified patients into low- and high-risk groups, with survival benefits up to 70% in the low-risk group. This approach offers a cost-effective and reproducible alternative to conventional molecular biomarkers, with external validation confirming its generalizability [41]. Additionally, Shi *et al*. [42] reported that radiomics signatures derived from pretreatment CE-T1WI MRI, when integrated with clinical factors such as the neutrophil-to-lymphocyte ratio and treatment lines, can predict intracranial response to ICIs in SCLC patients with brain metastases. Their nomogram showed stable predictive power for PFS and intracranial PFS, outperforming clinical factors alone. This suggests that combining radiomics features from both tumoral and peritumoral regions with key clinical markers can help tailor immunotherapy for SCLC patients with brain metastases. However, prospective studies with larger cohorts and standardized methods are still needed to confirm these findings.

### 4.3 Breast Cancer

Breast cancer is the second most common cancer globally and ranks fourth in cancer-related mortality [20]. For locally advanced disease, Neoadjuvant Chemotherapy (NAC) is standard, and its Pathological Complete Response (pCR) is an important prognostic marker [46]. Recently, immunotherapy has emerged as a promising option, especially when combined with chemotherapy to enhance tumor antigen release and modulate the immune microenvironment synergistically. Triple-Negative Breast Cancer (TNBC), defined by the lack of ER, PR, and HER2 expression, has limited treatment options and a poor prognosis, highlighting the need for immunotherapy. Notably, the FDA has approved atezolizumab combined with chemotherapy for PD-L1-positive unresectable or metastatic TNBC, marking the first approval of an immunotherapy for breast cancer.

In breast cancer patients, MRI is the most commonly employed auxiliary tool to evaluate tumor response to chemoimmunotherapy. MRI provides valuable insights into tumor shrinkage patterns and their correlation with therapeutic outcomes. After NAC, breast tumors exhibit varying degrees and patterns of contraction, with the NAC immunotherapy (NACI) group more likely to display a diffuse contraction pattern. Notably, all contraction patterns are associated with a high pCR rate [47]. In breast cancer research, DWI is extensively used to predict immunotherapy outcomes. By measuring the Apparent Diffusion Coefficient (ADC), DWI allows for the quantitative analysis of microstructural changes in tumor tissue. Studies have shown that high levels of TILs are often correlated with more homogeneous enhancement and higher ADC values [48]. Furthermore, some researchers have noted that, in patients receiving neoadjuvant immunotherapy, the mean ADC at early treatment stages outperforms functional tumor volume in predicting pCR [49]. Chemical Exchange Saturation Transfer (CEST) is a contrast agent technique that enables high-sensitivity, high-resolution detection of low-concentration endogenous metabolites. Hoffmann *et al*. [50] proposed a multi-parametric CEST-MRI technique to analyze the metabolic characteristics of two syngeneic mouse breast cancer models, evaluating whether these characteristics enable differentiation of the tumor microenvironment with varying degrees of malignancy. The experimental results demonstrated that multi-parametric CEST-MRI can non-invasively evaluate the metabolic characteristics of TME, estimating both tumor malignancy and early response to immune checkpoint inhibition.

Specific tumor and stromal imaging features are associated with TILs, and integrating imaging with molecular features may improve the prediction of TILs in breast cancer. Mammography, the preferred screening method for breast cancer, shows that radiomics-based quantitative features can distinguish TNBC from non-TNBC. In mammograms, TNBC with high TILs exhibits a lower histological grade than TNBC with low TILs [51]. Similarly, dynamic contrast-enhanced MRI also reveals a correlation between high TIL levels and a more aggressive tumor phenotype, characterized by larger tumor volume [52].

**Fig. (2):**
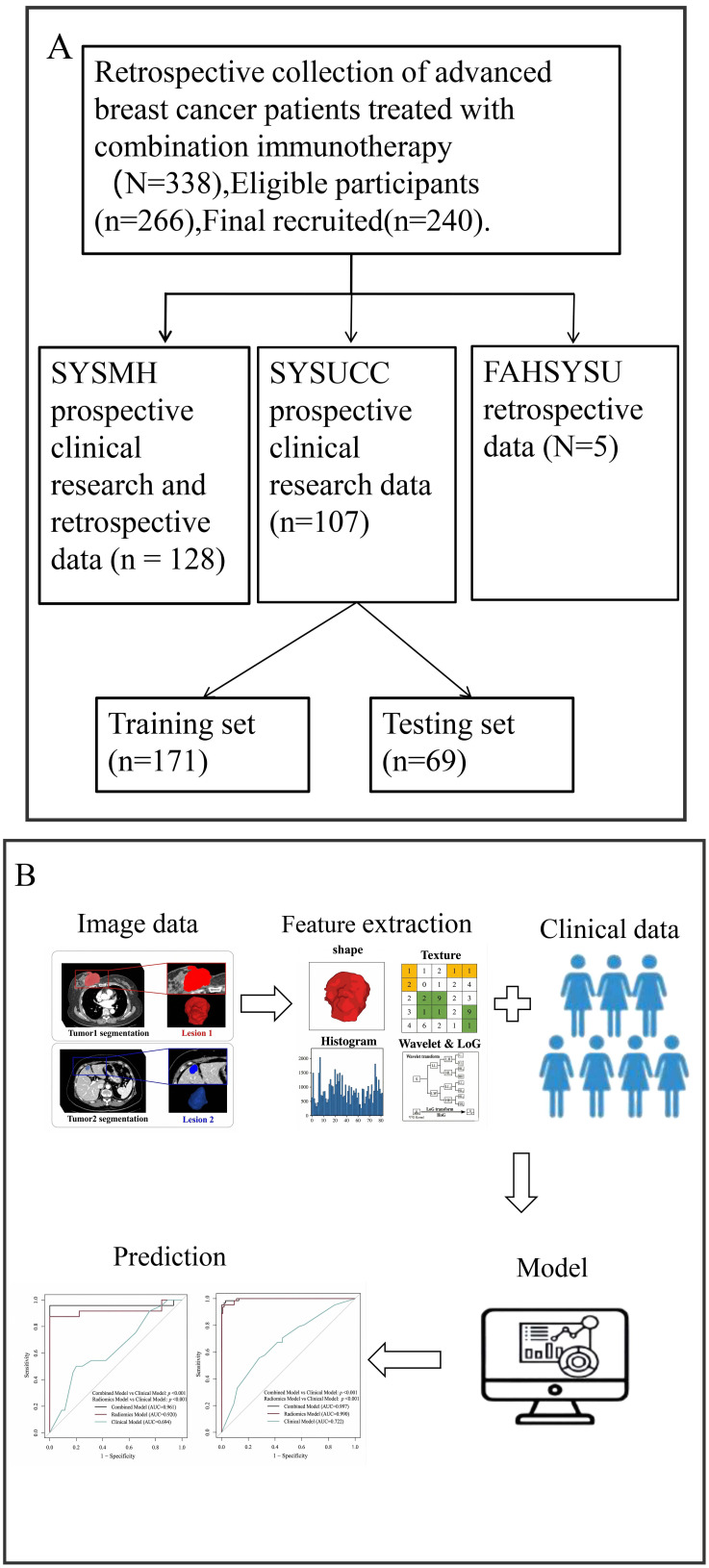
Schematic of experimental design (**A**) study participants. (**B**) Flow chart of the study steps.

Beyond TIL, numerous studies have investigated the association between radiomics and other immune-related biomarkers in breast cancer. Zhao *et al*. [53] developed a machine learning-based radiomic model specifically designed to predict the response to immunotherapy in patients with Advanced Breast Cancer (ABC) (Fig. **2**). This non-invasive predictive model performed effectively in both the training and validation cohorts, achieving AUCs of 0.994 and 0.920, respectively. Moreover, the radiomic model's accuracy remained unaffected by PD-L1 status, tumor metastasis burden, molecular subtype, or combination therapies. This radiomic model offers an innovative, accurate, and robust approach to stratifying ABC patients likely to benefit from ICI-based therapies, aiding in personalized treatment decisions. Han *et al*. [54] constructed and independently validated a biomarker for the tumor microenvironment phenotype using a machine learning-based radiomic approach in a cohort of 77 patients treated with anti-PD-1/PD-L1 therapy, achieving an AUC of 0.784. The results suggest that this imaging biomarker aids in predicting the TME phenotype and clinical response in breast cancer patients undergoing anti-PD-1/PD-L1 therapy. Additional studies related to BC are summarized in Table **4** [52-55].

### 4.4 Colorectal Cancer

Colorectal Cancer (CRC) is a prevalent malignancy, ranking third in global incidence (9.6%) and second in cancer-related mortality (9.3%) [20]. The diversity and heterogeneity of CRC have limited the development of effective treatment strategies beyond surgical resection in recent years. ICI therapy has emerged as a primary immunotherapy approach for CRC treatment. The feasibility and safety of ICIs in neoadjuvant therapy have been demonstrated across several tumor types, including CRC. In addition to ICIs, other emerging immunotherapies, such as Chimeric Antigen Receptor T (CAR-T) cell therapy and oncolytic virus-based treatments, have further enhanced the immunotherapy landscape for CRC. As a novel and potent anti-tumor strategy, immunotherapy is poised to become a viable alternative for CRC patients.

MSI in CRC involves defects in DNA mismatch repair, contributing to chemotherapy resistance but better immunotherapy responses [14]. Research reveals significant differences in metabolic parameters, tumor location, and age between MSI and MSS CRC patients. Liu *et al*. [56] identified MTV50% as an effective predictor of MSI-H, while Song *et al*. [57] validated that high MTV, younger age, and right-sided colon tumors are predictive of MSI-H. MSI-CRC, more common in individuals over 50, is associated with immune responses, with deep learning and digital pathology advancing outcome predictions. Golia *et al*. [58] and Fan *et al*. [59] successfully predicted MSI in colon cancer *via* preoperative CT radiomics, enhancing personalized treatment. MRI, superior in soft tissue contrast, predicts immune scores in rectal cancer when combining T2WI and ADC images [60, 61]. Additionally, ECT-based iodine MD imaging reflects angiogenesis, with IC values correlating with MSI status for non-invasive MSI assessment [62].

Hepatic and peritoneal metastases are prevalent in CRC, resulting in poorer prognoses and shorter survival. Studies have developed computer models to detect early liver metastasis. Texture analysis has shown promise for predicting CRC liver metastasis, while radiomics-enhanced CT models accurately identify MSI-H CRC with metastases [63]. Taghavi *et al*. [64] built a model predicting metachronous liver metastasis in low-risk patients. ^68^Ga FAPI-04 shows high tumor uptake in PET imaging, demonstrating its effectiveness for detecting intra-abdominal CRC metastases [65]. Li *et al*. [66] proposed dual PET/CT with ^68^Ga-FAPI and ^18^F-FDG to monitor metastatic CRC response, indicating it as a suitable CRC management tool. (Table **5**) [58, 60-63].

### 4.5 Gastric Cancer

Gastric cancer ranks fifth worldwide in both incidence and mortality [20]. Current immunotherapy mainly targets immune checkpoints. Pembrolizumab and Nivolumab have demonstrated clinical benefit in PD-L1-positive advanced gastric cancer and gained FDA approval for second-line or later treatment.

**Fig. (3):**
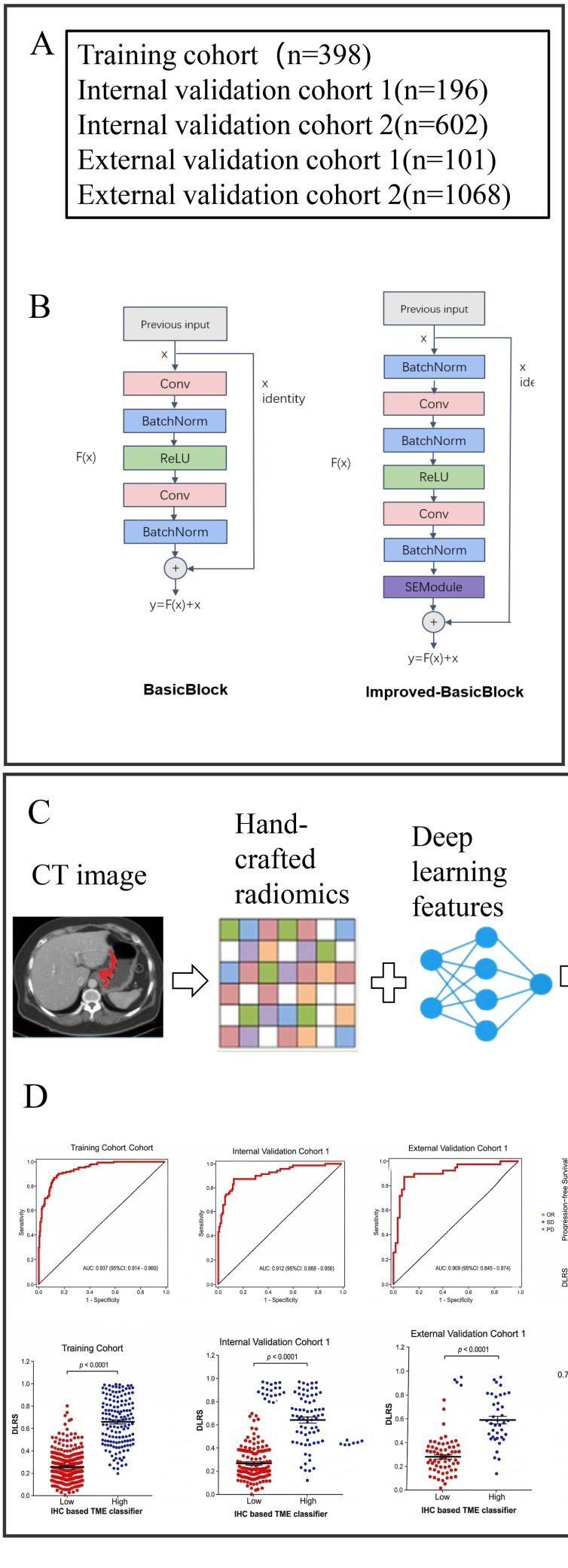
Schematic of Experimental Design (**A**) Study Participants. (**B**) The SE-based enhancement module integrates a channel attention mechanism, thereby enabling the network to more effectively capture implicit inter-channel information. (**C**) Flow chart of the study steps. (**D**) Performance Evaluation of the Deep Learning Model for Tumor Microenvironment Assessment in the Training Cohort, Internal Validation Cohort 1, and External Validation Cohort 1.

Radiomics, by extracting tumor texture features and other information, can predict response to immunotherapy in gastric cancer patients [67, 68]. Microsatellite Instability (MSI-H) or MMR deficiency is a significant biomarker in gastric cancer, closely related to patient survival. Patients with MSI have better overall survival than those with MSS [69], highlighting the importance of assessing MMR status in all gastric cancer patients as a Class I recommendation. Studies have shown a significant correlation between dMMR in gastric cancer and CT semantic features, with radiomics able to assess dMMR using CT-based features [70, 71]. In gastric cancer, patients with tumor infiltration by cytotoxic CD8 T lymphocytes tend to have longer survival, whereas those with high neutrophil content have a poorer prognosis [72]. Jiang *et al*. [73] trained a deep learning model using CT images to predict TME status, finding that a higher score from the model correlates with a better immunotherapy response (Fig. **3**). Sun *et al*. [74] developed lymphoid radiomics scores and myeloid radiomics scores to assess the immunological background, demonstrating that both scores serve as independent predictors of immunotherapy response. The Neutrophil-to-Lymphocyte Ratio (NLR) has been established as a prognostic indicator. Huang *et al*. [75] used CT radiomics scores to study the relationship between NLR and immunotherapy response, finding that radiomics scores align with IHC-derived NLR status and can predict patient PFS and OS, suggesting their potential as an alternative test for NLR.

Hayano *et al*. [76] recently conducted a retrospective study on patients with advanced gastric cancer undergoing immunotherapy, measuring the psoas muscle area before and after treatment. They calculated the skeletal Muscle Change Rate (MCR-SM) and Body Weight Change Rate (MCR-BW) to assess whether skeletal muscle changes before immunotherapy correlated with prognosis, and compared these findings with systemic inflammatory markers and liver lipid metabolism. They found that MCR-BW was not correlated with disease-specific survival or PFS, while MCR-SM was negatively correlated with NLR, MLR, and CRP, and positively correlated with LSR. The results showed that skeletal muscle loss before immunotherapy was linked to systemic inflammation, liver fat accumulation, and poorer prognosis in advanced gastric cancer patients. Preventing skeletal muscle loss during immunotherapy may improve treatment outcomes. Previously, Park *et al*. [77] noted that muscle mass decreases in AGC patients during first-line chemotherapy. Muscle mass decline, rather than weight or BMI reduction, is a key prognostic factor for gastric cancer patients during palliative chemotherapy. Fujii *et al*. [78] found that in 44 AGC patients treated with nivolumab, skeletal muscle loss was associated with poor clinical outcomes. Kano *et al*. [79] demonstrated that baseline psoas muscle mass index, a surrogate marker of skeletal muscle mass, predicts the response of advanced GC patients to nivolumab.

**Fig. (4):**
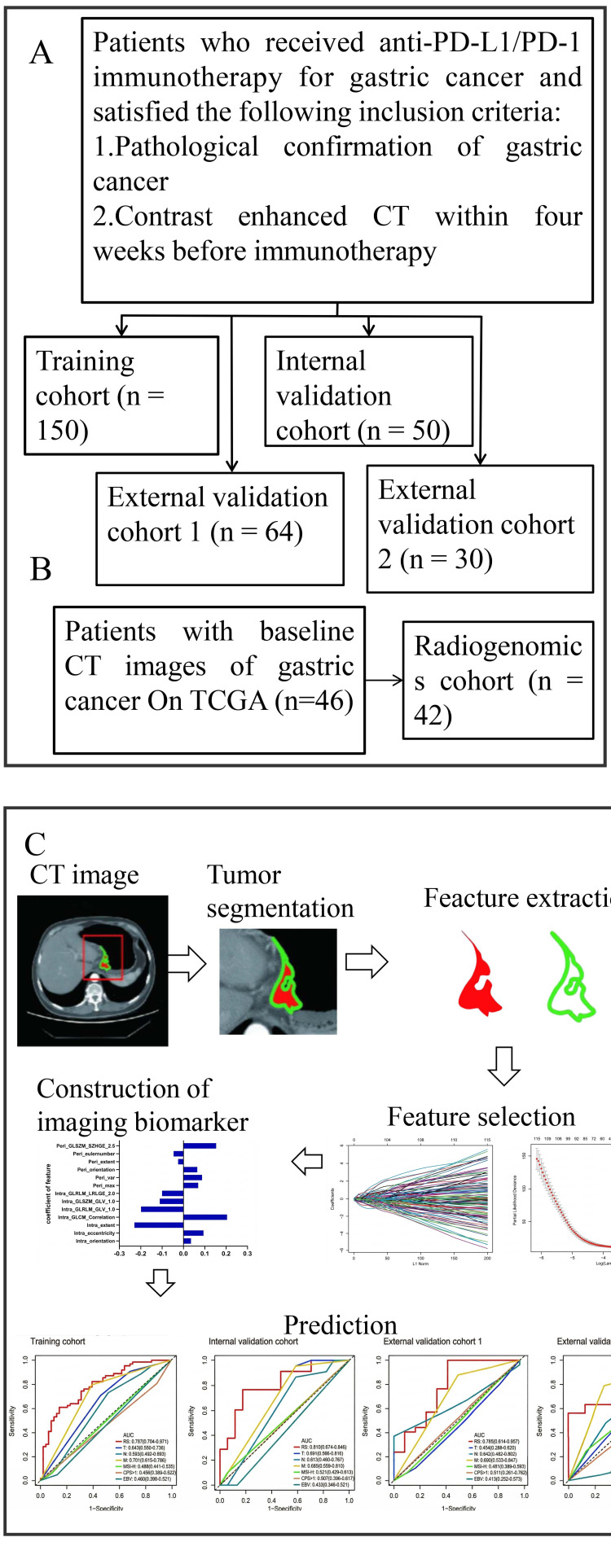
Schematic of Experimental Design (**A**) Flowchart of the Patient Recruitment Process. The retrospective study encompassed four cohorts from three academic medical centers in China. (**B**) Genomics sample. (**C**) Study Workflow Diagram. Tumor identification and segmentation were conducted on the largest cross-sectional slice of the primary tumor CT image. Intratumoral and peritumoral texture features were extracted and analyzed to identify the most predictive features for irPFS and to develop imaging biomarkers. The prognostic efficacy of these biomarkers was ultimately evaluated by predicting the response to immunotherapy.

Studies have shown that gastric cancer patients with high PD-L1 expression exhibit higher overall response rates than those with low PD-L1 expression [80]. The KEYNOTE-061 clinical trial found that patients with unresectable advanced or recurrent gastric cancer and a PD-L1 CPS > 10 are eligible for first-line PD-L1-targeted therapy [81]. Huang *et al*. [82] demonstrated that their non-invasive imaging biomarkers outperformed existing ones in predicting immunotherapy responses and were linked to innate immune signaling (Fig. **4**). Wang *et al*. [83] analyzed data from patients who underwent gastric surgery between 2019 and 2020, finding that advanced gastric cancer patients with CT features, such as strong arterial phase enhancement and large lymph nodes, were associated with a CPS ≥ 10. However, these features were not linked to an increase in immune cells. Chen *et al*. [84] were the first to investigate the relationship between ^18^F-FDG uptake and PD-L1/PD-L1-TILs expression in gastric cancer patients, finding that those with PD-L1 positivity had a higher SUVmax. However, while ^18^F-FDG-PET/CT may offer predictive value, the optimal SUVmax threshold for clinical practice remains undefined, and it cannot replace immunohistochemistry analysis. Hence, further prospective studies are needed to validate these findings. (Table **6**) [67, 68, 70, 71, 73-75, 82, 83, 85].

### 4.6 Malignant Melanoma

**Fig. (5):**
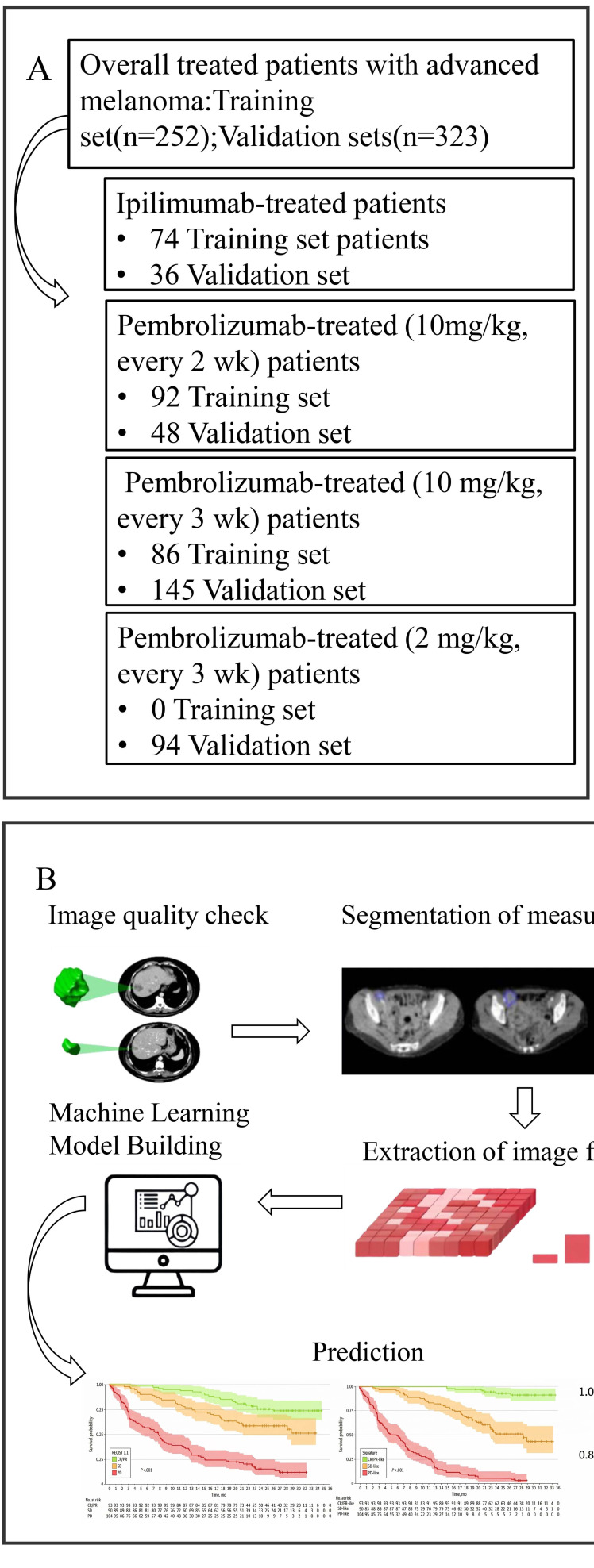
Schematic of experimental design (**A**) study participants. (**B**) Flow chart of the study steps.

**Fig. (6):**
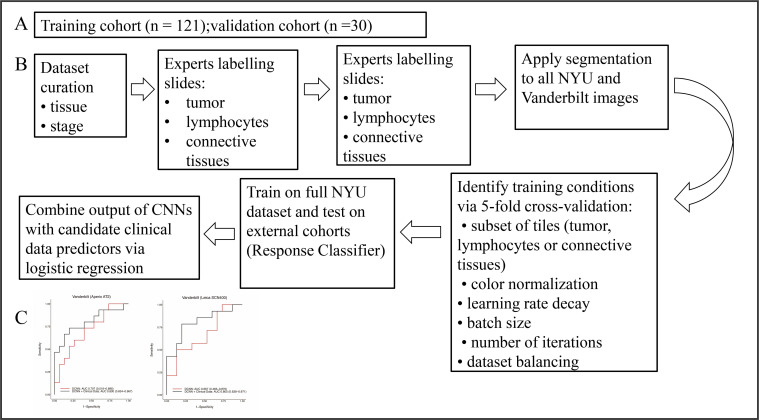
Schematic of experimental design (**A**) study participants. (**B**) Flow chart of the study steps. (**C**) Evaluation of the neural network and logistic regression classifiers was conducted using the ROC AUC.

Malignant melanoma, originating from skin melanocytes, accounted for approximately 331,000 new cases worldwide in 2022, with an incidence rate of 1.7% [20]. It is a highly aggressive cancer, ranking as the third most common skin cancer. Radiomics has identified biomarkers linked to serological (*e.g*., LDH) and molecular indicators (*e.g*., TMB), aiding in the diagnosis and treatment of advanced melanoma. Tabari *et al*. [86] found that integrating radiomics, machine learning, and LDH levels improves ICI efficacy prediction, enabling personalized treatment for metastatic melanoma. Feature selection has remained crucial in radiomics model building, as imaging software extracts many features, making dimensionality reduction techniques such as LASSO and logistic regression essential. Classifiers such as SVM and random forest are commonly applied after feature selection. In a study of 71 patients, Ungan *et al*. [87] identified Boruta feature selection with logistic regression as the most accurate for predicting treatment response and OS. CT texture analysis offers key biomarkers for predicting melanoma immunotherapy response. Dercle *et al*. [88] used a random forest model to construct radiomic features that accurately predicted overall survival in melanoma patients (Fig. **5**). Johannet *et al*. [89] developed a predictive model that integrates deep learning with clinical data to effectively differentiate between high- and low-risk groups in terms of immunotherapy outcomes (Fig. **6**). Durot *et al*. [90] found that pre-treatment CT texture skewness could predict OS and PFS in 31 patients. Schraag *et al*. [91] identified tumor burden and kurtosis as independent OS predictors in CT texture analysis. Basler *et al*. [92] indicated that PET/CT radiomics, combined with blood markers, is promising for early pseudo-progression differentiation, potentially reducing toxicity and avoiding premature treatment changes in metastatic melanoma patients on ICI therapy. Ayati *et al*. [93] found SUVpeak, MTV, and TLG promising for predicting immunotherapy response in their meta-analysis. (Table **7**) [87, 92, 94-96].

## 5 CURRENT CHALLENGES

Although significant advances have been made in evaluating the efficacy of tumor immunotherapy, several key challenges remain to its widespread clinical translation.

First, radiomics data are inherently high-dimensional and complex, and insufficient or imbalanced sample sizes can easily lead to model overfitting. Most radiomics models are still developed using retrospective, single-center datasets with limited external validation, which limits their generalizability in real-world clinical practice [97]. Additionally, multicenter datasets often lack harmonized processing; differences in imaging protocols, scanner equipment, and reconstruction parameters across institutions can compromise feature reproducibility and model robustness [98, 99]. The absence of standardized histopathological or molecular reference standards may introduce label noise, further limiting model training and validation performance. Moreover, patient privacy protection and data-sharing constraints remain major barriers to collaborative multicenter modeling.

Second, multi-modal and multi-phase workflows, such as combining PET/CT metabolic parameters with CT or MRI texture features, require highly precise image registration and standardized preprocessing. Respiratory motion artifacts, variations in patient positioning, or phase inconsistencies can introduce significant noise and reduce predictive accuracy. For example, delta-radiomics relies on strictly consistent imaging time points and treatment intervals to maintain longitudinal feature stability [100].

Regardless of the imaging modality, ROI segmentation remains a critical step, as most features depend on accurate delineation. Many studies still rely on manual tumor segmentation, which is operator-dependent and often inconsistent, thereby directly affecting the reliability of extracted features [101]. Although semi-automatic and fully automated segmentation tools have significantly improved feature stability and efficiency, the diversity of feature selection and dimensionality reduction methods (*e.g*., LASSO, Boruta, PCA) also affects reproducibility and comparability. Furthermore, significant differences in feature definitions, extraction algorithms, and parameter settings (such as gray-level bin width, filtering, or resampling) limit the cross-study consistency of handcrafted texture and delta-radiomics features [102].

Despite the substantial advances of deep learning and hybrid radiomics–AI frameworks in feature discovery and workflow automation, their “black-box” nature remains a major barrier to clinical translation. The opacity of decision-making processes limits clinicians' ability to verify model outputs, potentially undermining trust and adoption in routine practice. Explainable AI (XAI) offers a pathway to improve transparency by providing interpretable visual or quantitative representations of model reasoning, thereby enabling clinicians and patients to better understand and evaluate predictions. Moreover, XAI facilitates the evaluation of model architecture and decision-making processes, potentially contributing to biological discovery, for instance, by identifying molecular mechanisms underlying primary resistance to immunotherapy [103-105]. However, current XAI techniques are not yet standardized and often provide post hoc explanations that may not fully capture the underlying decision logic, underscoring the need for integrative, domain-specific interpretability approaches to facilitate safe and reliable clinical deployment.

## 6 DISCUSSION

Recent evidence underscores that radiomics workflows vary substantially by tumor type, imaging modality, biomarker integration, and analytic approach. In HCC, studies often employ multiphasic contrast-enhanced CT and multiparametric MRI to capture perfusion characteristics and assess microvascular invasion risk, with handcrafted features still favored for their interpretability. In lung cancer, PET/CT-derived metabolic metrics (*e.g*., SUVmax, MTV, TLG) are frequently combined with CT texture features, and CNN-based automated segmentation is increasingly adopted. Colorectal cancer radiomics is frequently integrated with molecular markers such as MSI status or the Immunoscore, while gastric cancer studies are beginning to incorporate PET metabolic imaging alongside multiphasic CT. In breast cancer, MRI provides superior soft-tissue contrast and functional sequences (*e.g*., DCE, DWI), enabling estimation of tumor-infiltrating lymphocytes and radiogenomic modeling; here, CNNs and Transformers are increasingly used for complex glandular segmentation. For melanoma, whole-body PET/CT is commonly used to monitor systemic metabolic alterations. Radiomics also differs by treatment context. In monotherapy, radiomic biomarkers are used to detect early immune response or resistance, based on features such as PD-L1 expression, tumor-infiltrating lymphocytes, and spatial heterogeneity in relatively homogeneous cohorts. In contrast, combination regimens involve more complex imaging signatures due to the interplay of multiple agents (*e.g*., anti-angiogenics that affect perfusion; chemotherapy that alters cellularity). These scenarios often require multimodal imaging to disentangle overlapping biological effects, increasing feature dimensionality and modeling complexity. Validation strategies diverge accordingly: monotherapy studies often rely on immune-related response criteria, whereas combination therapies require longitudinal imaging to assess synergistic or antagonistic effects. Tailoring radiomics pipelines to both tumor type and treatment setting is essential for developing robust, clinically translatable biomarkers.

In response to these challenges, cross-center collaborations and methodological innovations are helping address major barriers. Multi-center initiatives have begun to standardize acquisition protocols and harmonize preprocessing workflows, enhancing feature reproducibility and model transferability. For example, in lung cancer, hybrid pipelines that integrate PET metabolic parameters with CT radiomic features have demonstrated higher accuracy in predicting immunotherapy response. Previous studies have shown that in brain tumor and Alzheimer’s MRI classification, data complexity, heterogeneity, and class imbalance substantially affect the robustness and generalizability of deep learning models. However, strategies such as SMOTE oversampling, complexity assessment, and standardized preprocessing can improve model stability [106]. Multi-stage procedures, including advanced denoising, skull stripping, 3D reconstruction, and hybrid Transformer–U-Net segmentation, have also proven effective in reducing inter-modality variability and minimizing noise interference [107]. Deep learning-based semi-automatic or fully automatic segmentation tools are increasingly used to reduce operator dependency and ensure consistent ROI delineation. For instance, Khan *et al*. proposed a fully automated deep learning framework for segmentation and feature extraction that achieves high accuracy, supports survival prediction, and significantly reduces manual workload, demonstrating the potential of deep models for automation and clinical integration [107, 108]. Similarly, the D2PAM model for epilepsy prediction employs a dual-patch attention mechanism and a multi-network design to enhance feature extraction and model robustness [109]. In addition, explainable AI tools, such as heatmaps and attention maps, are being incorporated into CNN and Transformer architectures to improve biological interpretability and clinical acceptance. Notably, hybrid models that combine handcrafted radiomic features with deep learning representations hold promise for balancing interpretability and automated feature discovery.

To further accelerate clinical translation, future research should prioritize large-scale, prospective, externally validated multi-center studies. Additionally, standardized guidelines for segmentation, feature extraction, and reporting (*e.g*., RQS, TRIPOD) are needed. Integrating radiomics with molecular biomarkers and multi-omics data, together with advances in federated learning and privacy-preserving AI, may help expand clinical value while safeguarding patient privacy. Overall, developing robust, reproducible, and interpretable radiomics biomarkers will be key to driving innovation and achieving precision immunotherapy in real-world practice.

## CONCLUSION

As a potent non-invasive tool, radiomics has demonstrated promising potential for predicting and monitoring responses to immunotherapy and combination therapies across various solid tumors. This review comprehensively highlights that while radiomics workflows exhibit significant context-dependency, with imaging modalities, feature extraction, and analytic pipelines varying considerably across tumor types, this adaptability also represents a source of strength. Nevertheless, the urgent need for standardized frameworks, rigorous multi-center validation, and the development of robust, reproducible, and interpretable biomarkers remains paramount to enhance reproducibility and clinical reliability. Ultimately, integrating these advancements with advanced AI methodologies will be key to driving innovation and achieving seamless precision immunotherapy in real-world clinical practice.

## Figures and Tables

**Table 1:** Morphologic criteria for the assessment of response to immunotherapy.

**Assessment System**	**Item**			
RECIST1.1 (2009)	CR	PR	SD	PD
	Disappearance of all lesions	≥ 30% decrease from baseline	Neither CR nor PD	≥ 20% increase from the nadir (≥ 5mm)
irRC (2009)	irCR	irPR	irSD	irPD
	Disappearance of all lesions	≥ 50% decrease from baseline	Neither CR nor PD	≥ 25% increase from the nadir
irRECIST (2013)	irCR	irPR	irSD	irPD
	Disappearance of all lesions	≥ 30% decrease from baseline	Neither CR nor PD	≥ 20% increase from the nadir (≥ 5mm)
iRECIST (2017)	iCR	iPR	iSD	iCPD
	Disappearance of all lesions	≥ 30% decrease from baseline	Neither CR nor PD	≥ 20% increase from the nadir (≥ 5mm)
imRECIST (2018)	CR	PR	SD	PD
	Disappearance of all lesions	≥ 30% decrease from baseline	Neither CR nor PD	≥ 20% increase from the nadir (≥ 5mm)
itRECIST (2020)	CR	PR	SD	PD
	Disappearance of all lesions	≥ 30% decrease from last exam for injected lesions, ≥ 30% decrease from baseline for not injected lesions	Neither CR nor PD	≥ 20% increase from the nadir (≥ 5mm)
PERCIST (2009)	CMR	PMR	SMD	PMD
	Disappearance of all metabolically active lesions	Reduction (>30%, >0.8 unit) in SULpeak in the hottest target lesion	Neither PMD nor PMR/CMR	Increase (>30%, >0.8 unit) in SULpeak in the hottest target lesion, or New ^18^F-FDG-avid lesions
imPERCIST (2018)	CMR	PMR	SMD	PMD
	same as PERCIST	same as PERCIST	same as PERCIST	Increase (>30%, >0.8 unit) in SULpeak in the hottest target lesion. New ^18^F-FDG avid lesions alone do not conFig. PMD
Lugano (2014)	CR	PR	SD	PD
	Target lesions in lymph nodes with a long diameter of ≤1.5 cm, and the disappearance of extranodal lesions	A reduction of at least 50% in the product of the short and long diameters of up to 6 lymph nodes and extranodal lesions; when lesions are too small to be measured by CT, the size is uniformly set to 5×5 mm; when lesions are not visible, set to 0×0 mm; when the lymph node is larger than 5×5 mm, take the actual value	(1) Compared to the baseline, a reduction of <50% in the SPD of up to 6 measurable lymph nodes or extranodal lesions; (2) Does not meet the criteria for disease progression	At least one of the following: (1) Lesion diameter product (SPD) progression: at least one lesion with an LD ***i*** >1.5cm and an increase of at least 50% in SPD from the smallest value; (2) In patients with splenomegaly, an increase of more than 50% in spleen length from baseline; if the spleen was not enlarged at baseline, it must increase by at least 2.0cm; new or recurrent splenomegaly
LYRIC (2017)	CR	PR	SD	PD
	Same as the Lugano criteria	Same as the Lugano criteria	Lesion long diameter product shrinkage not reaching PR or increase not reaching PD.	Same as Lugano criteria, but excluding the following situations: Indeterminate response (IR): 1. Within the first 12 weeks, an increase of ≥50% in lesion SPD (based on the SPD of 6 measurable lesions), with no clinical deterioration. 2. At any time point after treatment, an increase of <50% in SPD, but accompanied by: a) the appearance of new lesions; b) an increase of ≥50% in the PPD of one or more lesions during treatment (based on the SPD of 6 measurable lesions), without an increase in the number of lesions. 3. An increase in lesion FDG uptake, without an increase or multiplication of the lesion itself.
RECIL (2017)	CR	PR	SD	PD
	Complete disappearance of all target lesions, with the longest diameter of all lymph nodes being < 10 mm	A decrease of at least 30% in the sum of the longest diameters of target lesions, but not reaching complete remission	A decrease of less than 10% or an increase of up to 20% in the sum of the longest diameters of target lesions	An increase of more than 20% in the sum of the longest diameters of target lesions; for lymph nodes with a diameter of <15 mm after treatment, they can only be classified as disease progression if the absolute increase in the long diameter is more than 5 mm and the total length is > 15 mm; the appearance of new lesions

**Table 2:** Existing literature on the use of radiomic methods to predict the efficacy of immunotherapy in HCC.

**Author/Year/Refs.**	**Modality**	**Biomarker**	**Method**	**Segmentation**	**Algorithm**	**N**	**AUC**	**Validation**
Chen *et al*., 2019 [32]	MRI	Immunoscore	​​Hybrid pipeline	Manual	Extra-Trees	207	0. 934	Internal
Tian *et al*.,2021 [33]	MRI	PD-L1 Expression	Hybrid pipeline	Manual	SVM	103	0. 897	Internal
Xin *et al*., 2023 [30]	CT	​​TIME	Handcrafted radiomics	Manual	LASSO regression	1133	0. 853	Internal
Wu *et al*., 2023 [31]	MRI	Immunoscore	Handcrafted radiomics	Manual	Ridge regression	301	0. 753	Internal

**Table 3:** Existing literature on the use of radiomic methods to predict the efficacy of immunotherapy in lung cancer.

**Author/Year/Refs.**	**Modality**	**Biomarker**	**Method**	**Segmentation**	**Algorithm**	**N**	**AUC**	**Validation**
Khorrami *et al*., 2020 [40]	CT	DelRADx, TIL	Handcrafted radiomics	Manual	LDA	139	0. 88	External multicenter
Zhao *et al*., 2023 [41]	CT	Radiomics score	Handcrafted radiomics	Manual	LASSO–Cox	341	0. 82	External multicenter
Da-ano *et al*., 2024 [36]	PET/CT	PD-L1	Hybrid pipeline	Semi-auto	CNN (ResNet, DenseNet, EfficientNet)	189	0. 84	Internal
Shi *et al*., 2024 [42]	MRI	ICI Response	Handcrafted radiomics	Manual	Random Forest,Logistic regression	101	0. 875	External multicenter

**Table 4:** Existing literature on the use of radiomic methods to predict the efficacy of immunotherapy in breast cancer.

**Author/Year/Refs.**	**Modality**	**Biomarker**	**Method**	**Segmentation**	**Algorithm**	**N**	**AUC**	**Validation**
Xu *et al*., 2020 [52]	MRI	TIL	Handcrafted radiomics	Manual	LASSO regression, Logistic regression	172	0. 842	Internal
Han *et al*., 2022 [55]	MRI	Immunoscore	Handcrafted radiomics	Manual	Random Forest	275	0. 815	External multicenter
Zhao *et al*., 2023 [53]	CT	ICI Response (CR/PR, SD/PD)	Handcrafted radiomics	Manual	MLP	240	0. 92	External multicenter
Han *et al*., 2024 [54]	MRI	TME	Handcrafted radiomics	Manual	Random Forest	1442	0. 844	external

**Table 5:** Existing literature on the use of radiomic methods to predict the efficacy of immunotherapy in colorectal cancer.

**Author/Year/Refs.**	**Modality**	**Biomarker**	**Method**	**Segmentation**	**Algorithm**	**N**	**AUC**	**Validation**
Pernicka *et al*.,2019 [58]	CT	MSI	Handcrafted radiomics	Manual	Random Forest	198	0. 79	Internal
Wu *et al*., 2019 [62]	CT	MSI	Handcrafted radiomics	Manual	LASSO regression, Logistic regression	102	0. 875	Internal
Li *et al*., 2021 [60]	MRI	MSI	Handcrafted radiomics	Manual	Random Forest, Logistic regression	90	0. 926	Internal
Xue *et al*., 2022 [61]	MRI	Immunoscore	Handcrafted radiomics	Manual	GBDT, Logistic regression	133	0. 768	Internal
Wang *et al*., 2023 [63]	CT	MSI	Handcrafted radiomics	Manual	Logistic regression	104	0. 858	Internal

**Table 6:** Existing literature on the use of radiomic methods to predict the efficacy of immunotherapy in gastric cancer.

**Author/Year/Refs.**	**Modality**	**Biomarker**	**Method**	**Segmentation**	**Algorithm**	**N**	**AUC**	**Validation**
Wang *et al*., 2022 [83]	CT	PD-L1 Expression	Handcrafted radiomics	Manual	Logistic regression	153	0. 671	Internal
Liang *et al*., 2022 [67]	CT	ICI Response (CR/PR, SD/PD)	Handcrafted radiomics	Manual	Logistic regression	87	0. 778	Internal
Zeng *et al*., 2022 [70]	CT	MMR deficiency (dMMR/pMMR)	Handcrafted radiomics	Manual	LASSO regression, Logistic regression	316	0. 891	external
Huang *et al*., 2022 [75]	CT	TIME, ICI response (ORR/PFS)	Hybrid pipeline	Manual	LASSO regression	2272	0. 861	External multicenter
Li *et al*., 2023 [68]	CT	PFS	Delta-radiomics	Manual	LASSO regression	42	C-index 0.705	Internal
Huang *et al*., 2023 [82]	CT	irPFS	Handcrafted radiomics,Delta-radiomics	Manual	LASSO–Cox	294	0. 81	Multicenter external
Chen *et al*., 2023 [71]	CT	dMMR	Handcrafted radiomics	Manual	Logistic regression	159	0. 895	Internal
Jiang *et al*., 2023 [73]	CT	TME	Hybrid pipeline	Manual	Modified HR-Net (SE)	2686	0. 909	External multicenter
Sun *et al*., 2024 [74]	CT	TIME	Handcrafted radiomics	Manual	LASSO logistic regression, SVM-RFE	2600	0. 78	External, prospective
Xu *et al*., 2024 [85]	CT	PD-L1 Expression (CPS ≥5 vs <5)	Handcrafted radiomics	Manual	ROC curve analysis	67	0. 825	Internal

**Table 7:** Existing literature on the use of radiomic methods to predict the efficacy of immunotherapy in melanoma.

**Author/Year/Refs.**	**Modality**	**Biomarker**	**Method**	**Segmentation**	**Algorithm**	**N**	**AUC**	**Validation**
Wang *et al*., 2020 [94]	CT	ICI Response (Poor/Good)	Hybrid pipeline	Manual	SVM, IMIA, Evidential Reasoning	50	0. 857	External
Basler *et al*., 2020 [92]	PET/CT	ICI Response (PP/TPD)	Delta-radiomics	Manual	Logistic regression, L2 regularization	112	0. 82	Internal
Chen *et al*., 2021 [95]	CT	ICI Response (Poor/Good)	Delta-radiomics	Manual	SVM with RBF kernel, Iterative Multi-objective Immune Algorithm	50	0. 86	External
Ungan *et al*., 2022 [87]	CT	Pretreatment CT texture	Handcrafted radiomics	Manual	Logistic regression	71	0. 88	Internal
Flaus *et al*., 2022 [96]	PET	Response (OS, PFS)	Handcrafted radiomics	Semi-automatic	Random Forest (RF)	56	0. 87-0.90	Internal

## LIST OF ABBREVIATIONS

WHO: World Health Organization
RECIST: Response Evaluation Criteria in Solid Tumors
NLR: Neutrophil-to-Lymphocyte Ratio
CT: Computed Tomography
PET: Positron Emission Tomography
MRI: Magnetic Resonance Imaging

## References

[1] Wolchok J.D., Hoos A., O’Day S., Weber J.S., Hamid O., Lebbé C., Maio M., Binder M., Bohnsack O., Nichol G., Humphrey R., Hodi F.S. (2009). Guidelines for the evaluation of immune therapy activity in solid tumors: Immune-related response criteria.. Clin. Cancer Res..

[2] Eisenhauer E.A., Therasse P., Bogaerts J., Schwartz L.H., Sargent D., Ford R., Dancey J., Arbuck S., Gwyther S., Mooney M., Rubinstein L., Shankar L., Dodd L., Kaplan R., Lacombe D., Verweij J. (2009). New response evaluation criteria in solid tumours: Revised RECIST guideline (version 1.1).. Eur. J. Cancer.

[3] Nishino M., Giobbie-Hurder A., Gargano M., Suda M., Ramaiya N.H., Hodi F.S. (2013). Developing a common language for tumor response to immunotherapy: Immune-related response criteria using unidimensional measurements.. Clin. Cancer Res..

[4] Seymour L., Bogaerts J., Perrone A., Ford R., Schwartz L.H., Mandrekar S., Lin N.U., Litière S., Dancey J., Chen A., Hodi F.S., Therasse P., Hoekstra O.S., Shankar L.K., Wolchok J.D., Ballinger M., Caramella C., de Vries E.G.E. (2017). iRECIST: Guidelines for response criteria for use in trials testing immunotherapeutics.. Lancet Oncol..

[5] Hodi FS., Ballinger M., Lyons B., Soria JC., Nishino M., Tabernero J., Powles T., Smith D., Hoos A., McKenna C., Beyer U., Rhee I., Fine G., Winslow N., Chen DS., Wolchok JD. (2018). Immune-modified response evaluation criteria in solid tumors (imRECIST): Refining guidelines to assess the clinical benefit of cancer immunotherapy.. J Clin Oncol.

[6] Goldmacher GV., Khilnani AD., Andtbacka RHI., Luke JJ., Hodi FS., Marabelle A., Harrington K., Perrone A., Tse A., Madoff DC., Schwartz LH. (2020). Response criteria for intratumoral immunotherapy in solid tumors: ItRECIST.. J Clin Oncol.

[7] Wahl R.L., Jacene H., Kasamon Y., Lodge M.A. (2009). From RECIST to PERCIST: Evolving Considerations for PET response criteria in solid tumors.. J. Nucl. Med..

[8] Ito K., Teng R., Schöder H., Humm JL., Ni A., Michaud L., Nakajima R., Yamashita R., Wolchok JD., Weber WA. (2019). 18F-FDG PET/CT for monitoring of ipilimumab therapy in patients with metastatic melanoma.. J Nucl Med.

[9] Cheson BD., Fisher RI., Barrington SF., Cavalli F., Schwartz LH., Zucca E., Lister TA. (2014). Recommendations for initial evaluation, staging, and response assessment of Hodgkin and non-Hodgkin lymphoma: The Lugano classification.. J Clin Oncol.

[10] Cheson B.D., Ansell S., Schwartz L., Gordon L.I., Advani R., Jacene H.A., Hoos A., Barrington S.F., Armand P. (2016). Refinement of the Lugano Classification lymphoma response criteria in the era of immunomodulatory therapy.. Blood.

[11] Younes A., Hilden P., Coiffier B., Hagenbeek A., Salles G., Wilson W., Seymour J.F., Kelly K., Gribben J., Pfreunschuh M., Morschhauser F., Schoder H., Zelenetz A.D., Rademaker J., Advani R., Valente N., Fortpied C., Witzig T.E., Sehn L.H., Engert A., Fisher R.I., Zinzani P.L., Federico M., Hutchings M., Bollard C., Trneny M., Elsayed Y.A., Tobinai K., Abramson J.S., Fowler N., Goy A., Smith M., Ansell S., Kuruvilla J., Dreyling M., Thieblemont C., Little R.F., Aurer I., Van Oers M.H.J., Takeshita K., Gopal A., Rule S., de Vos S., Kloos I., Kaminski M.S., Meignan M., Schwartz L.H., Leonard J.P., Schuster S.J., Seshan V.E. (2017). International Working Group consensus response evaluation criteria in lymphoma (RECIL 2017).. Ann. Oncol..

[12] Gillies R.J., Kinahan P.E., Hricak H. (2016). Radiomics: Images are more than pictures, they are data.. Radiology.

[13] Bruni D., Angell H.K., Galon J. (2020). The immune contexture and Immunoscore in cancer prognosis and therapeutic efficacy.. Nat. Rev. Cancer.

[14] Vilar E., Gruber S.B. (2010). Microsatellite instability in colorectal cancer—The stable evidence.. Nat. Rev. Clin. Oncol..

[15] van Timmeren J. E., Cester D., Tanadini-Lang S., Alkadhi H., Baessler B. (2020). Radiomics in medical imaging: "How-to" guide and critical reflection.. Insights Imaging.

[16] Dabass M., Vashisth S., Vig R. (2022). MTU: A multi-tasking U-net with hybrid convolutional learning and attention modules for cancer classification and gland segmentation in colon histopathological images.. Comput Biol Med.

[17] Qamar S., Ahmad P., Shen L. (2021). Dense encoder-decoder–based architecture for skin lesion segmentation.. Cognit. Comput..

[18] Li X., Lan Z., Sun Y., Sun Y., Guo Y., Wang Y., Yuan A. (2025). DPF-Unet: A CNN–Swin transformer fusion network for 3D brain tumor segmentation in MRI images.. J Supercomput.

[19] Kollem S., Peddakrishna S., Sirigiri C. An optimized GAN approach for denoising MRI brain tumor images.. 2024 4th International Conference on Artificial Intelligence and Signal Processing (AISP).

[20] Bray F., Laversanne M., Sung H., Ferlay J., Siegel R.L., Soerjomataram I., Jemal A. (2024). Global cancer statistics 2022: GLOBOCAN estimates of incidence and mortality worldwide for 36 cancers in 185 countries.. CA Cancer J. Clin..

[21] Yamashige D., Kawamura Y., Kobayashi M., Shindoh J., Kobayashi Y., Okubo S., Muraishi N., Kajiwara A., Iritani S., Fujiyama S., Hosaka T., Saitoh S., Sezaki H., Akuta N., Suzuki F., Suzuki Y., Ikeda K., Arase Y., Hashimoto M., Kumada H. (2021). Potential and clinical significance of 18F-Fluorodeoxyglucose positron emission tomography/computed tomography for evaluating liver cancer response to lenvatinib treatment.. Oncology.

[22] Kawamura Y., Kobayashi M., Shindoh J., Matsumura M., Okubo S., Muraishi N., Fujiyama S., Hosaka T., Saitoh S., Sezaki H., Akuta N., Suzuki F., Suzuki Y., Ikeda K., Arase Y., Hashimoto M., Kumada H. (2022). Pretreatment positron emission tomography with 18F-Fluorodeoxyglucose may be a useful new predictor of early progressive disease following atezolizumab plus bevacizumab in patients with unresectable hepatocellular carcinomama.. Oncology.

[23] Zhou X., Hu Y., Sun H., Chen R., Huang G., Liu J. (2023). Relationship between SUVmax on 18F-FDG PET and PD-L1 expression in hepatocellular carcinoma.. Eur. J. Nucl. Med. Mol. Imaging.

[24] Ueno A., Masugi Y., Yamazaki K., Kurebayashi Y., Tsujikawa H., Effendi K., Ojima H., Sakamoto M. (2020). Precision pathology analysis of the development and progression of hepatocellular carcinoma: Implication for precision diagnosis of hepatocellular carcinoma.. Pathol. Int..

[25] Kudo M. (2020). Gd-EOB-DTPA-MRI could predict WNT/β-catenin mutation and resistance to immune checkpoint inhibitor therapy in hepatocellular carcinoma.. Liver Cancer.

[26] Sasaki R., Nagata K., Fukushima M., Haraguchi M., Miuma S., Miyaaki H., Soyama A., Hidaka M., Eguchi S., Shigeno M., Yamashima M., Yamamichi S., Ichikawa T., Kugiyama Y., Yatsuhashi H., Nakao K. (2022). Evaluating the role of hepatobiliary phase of gadoxetic acid-enhanced magnetic resonance imaging in predicting treatment impact of lenvatinib and atezolizumab plus bevacizumab on unresectable hepatocellular carcinoma.. Cancers.

[27] Aoki T., Nishida N., Ueshima K., Morita M., Chishina H., Takita M., Hagiwara S., Ida H., Minami Y., Yamada A., Sofue K., Tsurusaki M., Kudo M. (2021). Higher enhancement intrahepatic nodules on the hepatobiliary phase of Gd-EOB-DTPA-enhanced MRI as a poor responsive marker of Anti-PD-1/PD-L1 monotherapy for unresectable hepatocellular carcinoma.. Liver Cancer.

[28] Sun L., Mu L., Zhou J., Tang W., Zhang L., Xie S., Chen J., Wang J. (2022). Imaging features of gadoxetic acid-enhanced MR imaging for evaluation of tumor-infiltrating CD8 cells and PD-L1 expression in hepatocellular carcinoma.. Cancer Immunol. Immunother..

[29] Yu G., Zhang Z., Eresen A., Hou Q., Garcia E.E., Yu Z., Abi-Jaoudeh N., Yaghmai V., Zhang Z. (2024). MRI radiomics to monitor therapeutic outcome of sorafenib plus IHA transcatheter NK cell combination therapy in hepatocellular carcinoma.. J. Transl. Med..

[30] Xin H., Lai Q., Zhou Y., He J., Song Y., Liao M., Sun J., Li M., Zhang M., Liang W., Bai Y., Zhang Y., Zhou Y. (2023). Noninvasive evaluation of neutrophil extracellular traps signature predicts clinical outcomes and immunotherapy response in hepatocellular carcinoma.. Front. Immunol..

[31] Wu J., Liu W., Qiu X., Li J., Song K., Shen S., Huo L., Chen L., Xu M., Wang H., Jia N., Chen L. (2023). A noninvasive approach to evaluate tumor immune microenvironment and predict outcomes in hepatocellular carcinoma.. Phenomics.

[32] Chen S., Feng S., Wei J., Liu F., Li B., Li X., Hou Y., Gu D., Tang M., Xiao H., Jia Y., Peng S., Tian J., Kuang M. (2019). Pretreatment prediction of immunoscore in hepatocellular cancer: A radiomics-based clinical model based on Gd-EOB-DTPA-enhanced MRI imaging.. Eur. Radiol..

[33] Tian Y., Komolafe T.E., Zheng J., Zhou G., Chen T., Zhou B., Yang X. (2021). Assessing PD-L1 expression level *via* preoperative MRI in HCC based on integrating deep learning and radiomics features.. Diagnostics.

[34] Kazandjian D., Suzman D.L., Blumenthal G., Mushti S., He K., Libeg M., Keegan P., Pazdur R. (2016). FDA approval summary: Nivolumab for the treatment of metastatic non-small cell lung cancer with progression on or after platinum-based chemotherapy.. Oncologist.

[35] Mu W., Jiang L., Shi Y., Tunali I., Gray JE., Katsoulakis E., Tian J., Gillies RJ., Schabath MB. (2021). Non-invasive measurement of PD-L1 status and prediction of immunotherapy response using deep learning of PET/CT images.. J Immunother Cancer.

[36] Da-Ano R., Andrade-Miranda G., Tankyevych O., Visvikis D., Conze P.-H., Rest C. C. L. (2024). Automated PD-L1 status prediction in lung cancer with multi-modal PET/CT fusion.. Sci Rep.

[37] Chen M., Lu H., Copley S.J., Han Y., Logan A., Viola P., Cortellini A., Pinato D.J., Power D., Aboagye E.O. (2023). A novel radiogenomics biomarker for predicting treatment response and pneumotoxicity from programmed cell death protein or Ligand-1 inhibition immunotherapy in NSCLC.. J. Thorac. Oncol..

[38] Seban R.D., Mezquita L., Berenbaum A., Dercle L., Botticella A., Le Pechoux C., Caramella C., Deutsch E., Grimaldi S., Adam J., Ammari S., Planchard D., Leboulleux S., Besse B. (2020). Baseline metabolic tumor burden on FDG PET/CT scans predicts outcome in advanced NSCLC patients treated with immune checkpoint inhibitors.. Eur. J. Nucl. Med. Mol. Imaging.

[39] Kudura K., Ritz N., Kutzker T., Hoffmann M.H.K., Templeton A.J., Foerster R., Kreissl M.C., Antwi K. (2022). Predictive value of baseline FDG-PET/CT for the durable response to immune checkpoint inhibition in NSCLC patients using the morphological and metabolic features of primary tumors.. Cancers.

[40] Khorrami M., Prasanna P., Gupta A., Patil P., Velu P.D., Thawani R., Corredor G., Alilou M., Bera K., Fu P., Feldman M., Velcheti V., Madabhushi A. (2020). Changes in CT radiomic features associated with lymphocyte distribution predict overall survival and response to immunotherapy in non–small cell lung cancer.. Cancer Immunol. Res..

[41] Zhao J., He Y., Yang X., Tian P., Zeng L., Huang K., Zhao J., Zhou J., Zhu Y., Wang Q., Chen M., Li W., Gao Y., Zhang Y., Xia Y. (2023). Assessing treatment outcomes of chemoimmunotherapy in extensive-stage small cell lung cancer: An integrated clinical and radiomics approach.. J. Immunother. Cancer.

[42] Shi X., Wang P., Li Y., Xu J., Yin T., Teng F. (2024). Using MRI radiomics to predict the efficacy of immunotherapy for brain metastasis in patients with small cell lung cancer.. Thorac. Cancer.

[43] Martucci F., Pascale M., Valli MC., Pesce GA., Froesch P., Giovanella L., Richetti A., Treglia G. (2020). Impact of 18F-FDG PET/CT in staging patients with small cell lung cancer: A systematic review and meta-analysis.. Front Med.

[44] Zer A., Domachevsky L., Rapson Y., Nidam M., Flex D., Allen A.M., Stemmer S.M., Groshar D., Bernstine H. (2016). The role of 18F-FDG PET/CT on staging and prognosis in patients with small cell lung cancer.. Eur. Radiol..

[45] Grambow-Velilla J., Seban R.D., Chouahnia K., Assié J.B., Champion L., Girard N., Bonardel G., Matton L., Soussan M., Chouaïd C., Duchemann B. (2023). Total metabolic tumor volume on 18F-FDG PET/CT is a useful prognostic biomarker for patients with extensive small-cell lung cancer undergoing first-line chemo-immunotherapy.. Cancers.

[46] Thompson A.M., Moulder-Thompson S.L. (2012). Neoadjuvant treatment of breast cancer.. Ann. Oncol..

[47] Zou J., Zhang L., Chen Y., Lin Y., Cheng M., Zheng X., Zhuang X., Wang K. (2024). Neoadjuvant chemotherapy and neoadjuvant chemotherapy with immunotherapy result in different tumor shrinkage patterns in triple-negative breast cancer.. J. Breast Cancer.

[48] Çelebi F., Agacayak F., Ozturk A., Ilgun S., Ucuncu M., Iyigun Z.E., Ordu Ç., Pilanci K.N., Alco G., Gultekin S., Cindil E., Soybir G., Aktepe F., Özmen V. (2020). Usefulness of imaging findings in predicting tumor-infiltrating lymphocytes in patients with breast cancer.. Eur. Radiol..

[49] Li W., Le N.N., Onishi N., Newitt D.C., Wilmes L.J., Gibbs J.E., Carmona-Bozo J., Liang J., Partridge S.C., Price E.R., Joe B.N., Kornak J., Magbanua M.J.M., Nanda R., LeStage B., Esserman L.J., van’t Veer L.J., Hylton N.M. (2022). Diffusion-weighted MRI for predicting pathologic complete response in neoadjuvant immunotherapy.. cancers.

[50] Hoffmann E., Schache D., Höltke C., Soltwisch J., Niland S., Krähling T., Bergander K., Grewer M., Geyer C., Groeneweg L., Eble J.A., Vogl T., Roth J., Heindel W., Maus B., Helfen A., Faber C., Wildgruber M., Gerwing M., Hoerr V. (2023). Multiparametric chemical exchange saturation transfer MRI detects metabolic changes in breast cancer following immunotherapy.. J. Transl. Med..

[51] Yu H., Meng X., Chen H., Han X., Fan J., Gao W., Du L., Chen Y., Wang Y., Liu X., Zhang L., Ma G., Yang J. (2020). Correlation between mammographic radiomics features and the level of tumor-infiltrating lymphocytes in patients with triple-negative breast cancer.. Front. Oncol..

[52] Xu N., Zhou J., He X., Ye S., Miao H., Liu H., Chen Z., Zhao Y., Pan Z., Wang M. (2021). Radiomics model for evaluating the level of tumor-infiltrating lymphocytes in breast cancer based on dynamic contrast-enhanced MRI.. Clin Breast Cancer.

[53] Zhao J., Sun Z., Yu Y., Yuan Z., Lin Y., Tan Y., Duan X., Yao H., Wang Y., Liu J. (2023). Radiomic and clinical data integration using machine learning predict the efficacy of anti-PD-1 antibodies-based combinational treatment in advanced breast cancer: A multicentered study.. J. Immunother. Cancer.

[54] Han X., Guo Y., Ye H., Chen Z., Hu Q., Wei X., Liu Z., Liang C. (2024). Development of a machine learning-based radiomics signature for estimating breast cancer TME phenotypes and predicting anti-PD-1/PD-L1 immunotherapy response.. Breast Cancer Res..

[55] Han X., Cao W., Wu L., Liang C. (2022). Radiomics assessment of the tumor immune microenvironment to predict outcomes in breast cancer.. Front. Immunol..

[56] Liu H., Ye Z., Yang T., Xie H., Duan T., Li M., Wu M., Song B. (2021). Predictive value of metabolic parameters derived from 18F-FDG PET/CT for microsatellite instability in patients with colorectal carcinoma.. Front Immunol.

[57] Song J., Li Z., Yang L., Wei M., Yang Z., Wang X. (2022). Metabolic activity *via*
^18^F-FDG PET/CT is predictive of microsatellite instability status in colorectal cancer.. BMC Cancer.

[58] Golia Pernicka J.S., Gagniere J., Chakraborty J., Yamashita R., Nardo L., Creasy J.M., Petkovska I., Do R.R.K., Bates D.D.B., Paroder V., Gonen M., Weiser M.R., Simpson A.L., Gollub M.J. (2019). Radiomics-based prediction of microsatellite instability in colorectal cancer at initial computed tomography evaluation.. Abdom. Radiol..

[59] Fan S., Li X., Cui X., Zheng L., Ren X., Ma W., Ye Z. (2019). Computed tomography-based radiomic features could potentially predict microsatellite instability status in stage II colorectal cancer: A preliminary study.. Acad. Radiol..

[60] Li Z., Dai H., Liu Y., Pan F., Yang Y., Zhang M. (2021). Radiomics analysis of multi-sequence MR images for predicting microsatellite instability status preoperatively in rectal cancer.. Front. Oncol..

[61] Xue K., Liu L., Liu Y., Guo Y., Zhu Y., Zhang M. (2022). Radiomics model based on multi-sequence MR images for predicting preoperative immunoscore in rectal cancer.. Radiol. Med..

[62] Wu J., Zhang Q., Zhao Y., Liu Y., Chen A., Li X., Wu T., Li J., Guo Y., Liu A. (2019). Radiomics analysis of iodine-based material decomposition images with dual-energy computed tomography imaging for preoperatively predicting microsatellite instability status in colorectal cancer.. Front. Oncol..

[63] Wang X., Liu Z., Yin X., Yang C., Zhang J. (2023). A radiomics model fusing clinical features to predict microsatellite status preoperatively in colorectal cancer liver metastasis.. BMC Gastroenterol..

[64] Taghavi M., Trebeschi S., Simões R., Meek D.B., Beckers R.C.J., Lambregts D.M.J., Verhoef C., Houwers J.B., van der Heide U.A., Beets-Tan R.G.H., Maas M. (2021). Machine learning-based analysis of CT radiomics model for prediction of colorectal metachronous liver metastases.. Abdom. Radiol..

[65] Chen H., Pang Y., Wu J., Zhao L., Hao B., Wu J., Wei J., Wu S., Zhao L., Luo Z., Lin X., Xie C., Sun L., Lin Q., Wu H. (2020). Comparison of [68Ga]Ga-DOTA-FAPI-04 and [18F] FDG PET/CT for the diagnosis of primary and metastatic lesions in patients with various types of cancer.. Eur J Nucl Med Mol Imaging.

[66] Li K., Liu W., Yu H., Chen J., Tang W., Wang J., Qi M., Sun Y., Xu X., Zhang J., Li X., Guo W., Li X., Song S., Tang S. (2024). 68Ga-FAPI PET imaging monitors response to combined TGF-βR inhibition and immunotherapy in metastatic colorectal cancer.. J Clin Invest.

[67] Liang Z., Huang A., Wang L., Bi J., Kuang B., Xiao Y., Yu D., Hong M., Zhang T. (2022). A radiomics model predicts the response of patients with advanced gastric cancer to PD-1 inhibitor treatment.. Aging.

[68] Li J., Chen Z., Chen Y., Zhao J., He M., Li X., Zhang L., Dong B., Zhang X., Tang L., Shen L. (2023). CT-based delta radiomics in predicting the prognosis of stage IV gastric cancer to immune checkpoint inhibitors.. Front. Oncol..

[69] Pietrantonio F., Randon G., Di Bartolomeo M., Luciani A., Chao J., Smyth E.C., Petrelli F. (2021). Predictive role of microsatellite instability for PD-1 blockade in patients with advanced gastric cancer: A meta-analysis of randomized clinical trials.. ESMO Open.

[70] Zeng Q., Zhu Y., Li L., Feng Z., Shu X., Wu A., Luo L., Cao Y., Tu Y., Xiong J., Zhou F., Li Z. (2022). CT-based radiomic nomogram for preoperative prediction of DNA mismatch repair deficiency in gastric cancer.. Front. Oncol..

[71] Chen J.Y., Tong Y.H., Chen H.Y., Yang Y.B., Deng X.Y., Shao G.L. (2023). A noninvasive nomogram model based on CT features to predict DNA mismatch repair deficiency in gastric cancer.. Front. Oncol..

[72] Jiang Y., Xie J., Han Z., Liu W., Xi S., Huang L., Huang W., Lin T., Zhao L., Hu Y., Yu J., Zhang Q., Li T., Cai S., Li G. (2018). Immunomarker support vector machine classifier for prediction of gastric cancer survival and adjuvant chemotherapeutic benefit.. Clin. Cancer Res..

[73] Jiang Y., Zhou K., Sun Z., Wang H., Xie J., Zhang T., Sang S., Islam M.T., Wang J.Y., Chen C., Yuan Q., Xi S., Li T., Xu Y., Xiong W., Wang W., Li G., Li R. (2023). Non-invasive tumor microenvironment evaluation and treatment response prediction in gastric cancer using deep learning radiomics.. Cell Rep. Med..

[74] Sun Z., Zhang T., Ahmad M.U., Zhou Z., Qiu L., Zhou K., Xiong W., Xie J., Zhang Z., Chen C., Yuan Q., Chen Y., Feng W., Xu Y., Yu L., Wang W., Yu J., Li G., Jiang Y. (2024). Comprehensive assessment of immune context and immunotherapy response *via* noninvasive imaging in gastric cancer.. J. Clin. Invest..

[75] Huang W., Jiang Y., Xiong W., Sun Z., Chen C., Yuan Q., Zhou K., Han Z., Feng H., Chen H., Liang X., Yu S., Hu Y., Yu J., Chen Y., Zhao L., Liu H., Zhou Z., Wang W., Wang W., Xu Y., Li G. (2022). Noninvasive imaging of the tumor immune microenvironment correlates with response to immunotherapy in gastric cancer.. Nat. Commun..

[76] Hayano K., Ohira G., Matsumoto Y., Kurata Y., Otsuka R., Hirata A., Toyozumi T., Murakami K., Uesato M., Matsubara H. (2024). CT-derived skeletal muscle change before immunotherapy predicts survival of advanced gastric cancer: Associations with inflammatory markers and liver lipid metabolism.. Int. J. Clin. Oncol..

[77] Park S.E., Choi J.H., Park J.Y., Kim B.J., Kim J.G., Kim J.W., Park J.M., Chi K.C., Hwang I.G. (2020). Loss of skeletal muscle mass during palliative chemotherapy is a poor prognostic factor in patients with advanced gastric cancer.. Sci. Rep..

[78] Fujii H., Makiyama A., Iihara H., Okumura N., Yamamoto S., Imai T., Arakawa S., Kobayashi R., Tanaka Y., Yoshida K., Suzuki A. (2020). Cancer cachexia reduces the efficacy of nivolumab treatment in patients with advanced gastric cancer.. Anticancer Res..

[79] Kano M., Hihara J., Tokumoto N., Kohashi T., Hara T., Shimbara K., Takahashi S. (2021). Association between skeletal muscle loss and the response to nivolumab immunotherapy in advanced gastric cancer patients.. Int. J. Clin. Oncol..

[80] Kim S.T., Cristescu R., Bass A.J., Kim K.M., Odegaard J.I., Kim K., Liu X.Q., Sher X., Jung H., Lee M., Lee S., Park S.H., Park J.O., Park Y.S., Lim H.Y., Lee H., Choi M., Talasaz A., Kang P.S., Cheng J., Loboda A., Lee J., Kang W.K. (2018). Comprehensive molecular characterization of clinical responses to PD-1 inhibition in metastatic gastric cancer.. Nat Med.

[81] Shitara K., Özgüroğlu M., Bang Y.J., Di Bartolomeo M., Mandalà M., Ryu M.H., Fornaro L., Olesiński T., Caglevic C., Chung H.C., Muro K., Goekkurt E., Mansoor W., McDermott R.S., Shacham-Shmueli E., Chen X., Mayo C., Kang S.P., Ohtsu A., Fuchs C.S., Lerzo G., O’Connor J.M., Mendez G.A., Lynam J., Tebbutt N., Wong M., Strickland A., Karapetis C., Goldstein D., Vasey P., Van Laethem J-L., Van Cutsem E., Berry S., Vincent M., Muller B., Rey F., Zambrano A., Guerra J., Krogh M., Baeksgaard L., Yilmaz M., Elme A., Magi A., Auvinen P., Alanko T., Moehler M., Kunzmann V., Seufferlein T., Thuss-Patience P., Goekkurt E., Hoehler T., Haag G., Al-Batran S-E., Castro H., Lopez K., Aguilar Vasquez M., Sandoval M., Lam K.O., Cuffe S., Kelly C., Geva R., Shacham-Shmueli E., Hubert A., Beny A., Brenner B., Giuseppe A., Falcone A., Maiello E., Passalacqua R., Montesarchio V., Hara H., Chin K., Nishina T., Komatsu Y., Machida N., Hironaka S., Satoh T., Tamura T., Sugimoto N., Cho H., Omuro Y., Kato K., Goto M., Hyodo I., Yoshida K., Baba H., Esaki T., Furuse J., Wan Mohammed W.Z., Hernandez Hernandez C., Casas Garcia J., Dominguez Andrade A., Clarke K., Hjortland G., Glenjen N., Kubiatowski T., Jacek J., Wojtukiewicz M., Lazarev S., Lancukhay Y., Afanasayev S., Moiseyenko V., Kostorov V., Protsenko S., Shirinkin V., Sakaeva D., Fadeeva N., Yong W.P., Ng C.H.M., Robertson B., Rapaport B., Cohen G., Dreosti L., Ruff P., Jacobs C., Landers G., Szpak W., Roh S-Y., Lee J., Kim Y.H., Bang Y-J., Chung H.C., Ryu M-H., Alsina Maqueda M., Longo Munoz F., Cervantes Aguilar A., Aranda Aguilar E., Garcia Alfonso P., Rivera F., Feliu Batle J., Pazo Cid R., Yeh K-H., Chen J-S., Chao Y., Yen C-J., Özgüroğlu M., Kara O., Yalcin S., Hochhauser D., Chau I., Benson A., Shankaran V., Shaib W., Philip P., Sharma V., Siegel R., Sun W., Wainberg Z., George B., Bullock A., Myrick S., Faruol J., Siegel R., Larson T., Becerra C., Ratnam S., Richards D.A., Riche S.L. (2018). Pembrolizumab versus paclitaxel for previously treated, advanced gastric or gastro-oesophageal junction cancer (KEYNOTE-061): A randomised, open-label, controlled, phase 3 trial.. Lancet.

[82] Huang W., Xiong W., Tang L., Chen C., Yuan Q., Zhang C., Zhou K., Sun Z., Zhang T., Han Z., Feng H., Liang X., Zhong Y., Deng H., Yu L., Xu Y., Wang W., Shen L., Li G., Jiang Y. (2023). Non-invasive CT imaging biomarker to predict immunotherapy response in gastric cancer: A multicenter study.. J. Immunother. Cancer.

[83] Wang Z., Wang Y., Li X., Li Y., Bu Z., Li Z., Sun Y., Ji J. (2022). Correlation between imaging features on computed tomography and combined positive score of PD-L1 expression in patients with gastric cancer.. Chin. J. Cancer Res..

[84] Chen R., Chen Y., Huang G., Liu J. (2019). Relationship between PD-L1 expression and ^18^F-FDG uptake in gastric cancer.. Aging.

[85] Xu M., Ren T., Deng J., Yang J., Lu T., Xi H., Yuan L., Zhang W., Zhou J. (2024). Correlation of CT parameters and PD-L1 expression status in gastric cancer.. Abdom Radiol.

[86] Tabari A., Cox M., D’Amore B., Mansur A., Dabbara H., Boland G., Gee M.S., Daye D. (2023). Machine Learning improves the prediction of responses to immune checkpoint inhibitors in metastatic melanoma.. Cancers.

[87] Ungan G., Lavandier A.F., Rouanet J., Hordonneau C., Chauveau B., Pereira B., Boyer L., Garcier J.M., Mansard S., Bartoli A., Magnin B. (2022). Metastatic melanoma treated by immunotherapy: Discovering prognostic markers from radiomics analysis of pretreatment CT with feature selection and classification.. Int. J. CARS.

[88] Dercle L., Zhao B., Gönen M., Moskowitz C.S., Firas A., Beylergil V., Connors D.E., Yang H., Lu L., Fojo T., Carvajal R., Karovic S., Maitland M.L., Goldmacher G.V., Oxnard G.R., Postow M.A., Schwartz L.H. (2022). Early readout on overall survival of patients with melanoma treated with immunotherapy using a novel imaging analysis.. JAMA Oncol..

[89] Johannet P., Coudray N., Donnelly D.M., Jour G., Illa-Bochaca I., Xia Y., Johnson D.B., Wheless L., Patrinely J.R., Nomikou S., Rimm D.L., Pavlick A.C., Weber J.S., Zhong J., Tsirigos A., Osman I. (2021). Using machine learning algorithms to predict immunotherapy response in patients with advanced melanoma.. Clin Cancer Res.

[90] Durot C., Mulé S., Soyer P., Marchal A., Grange F., Hoeffel C. (2019). Metastatic melanoma: Pretreatment contrast-enhanced CT texture parameters as predictive biomarkers of survival in patients treated with pembrolizumab.. Eur. Radiol..

[91] Schraag A., Klumpp B., Afat S., Gatidis S., Nikolaou K., Eigentler T.K., Othman A.E. (2019). Baseline clinical and imaging predictors of treatment response and overall survival of patients with metastatic melanoma undergoing immunotherapy.. Eur. J. Radiol..

[92] Basler L., Gabryś H.S., Hogan S.A., Pavic M., Bogowicz M., Vuong D., Tanadini-Lang S., Förster R., Kudura K., Huellner M.W., Dummer R., Guckenberger M., Levesque M.P. (2020). Radiomics, tumor volume, and blood biomarkers for early prediction of pseudoprogression in patients with metastatic melanoma treated with immune checkpoint inhibition.. Clin. Cancer Res..

[93] Ayati N., Sadeghi R., Kiamanesh Z., Lee S.T., Zakavi S.R., Scott A.M. (2021). The value of 18F-FDG PET/CT for predicting or monitoring immunotherapy response in patients with metastatic melanoma: A systematic review and meta-analysis.. Eur J Nucl Med Mol Imaging.

[94] Wang Z., Mao L., Zhou Z., Si L., Zhu H., Chen X., Zhou M., Sun Y., Guo J. (2020). Pilot study of CT-based radiomics model for early evaluation of response to immunotherapy in patients with metastatic melanoma.. Front. Oncol..

[95] Chen X., Zhou M. J., Wang Z. L., Lu S., Chang S. J., Zhou Z. G. (2021). Immunotherapy treatment outcome prediction in metastatic melanoma through an automated multi-objective delta-radiomics model.. Comput Biol Med.

[96] Flaus A., Habouzit V., de Leiris N., Vuillez JP., Leccia MT., Simonson M., Perrot JL., Cachin F., Prevot N. (2022). Outcome prediction at patient level derived from pre-treatment 18F-FDG PET due to machine learning in metastatic melanoma treated with anti-PD1 treatment.. Diagnostics.

[97] Chalkidou A., O'Doherty MJ., Marsden PK. (2015). False discovery rates in PET and CT studies with texture features: A systematic review.. PLoS One.

[98] Zwanenburg A. (2019). Radiomics in nuclear medicine: Robustness, reproducibility, standardization, and how to avoid data analysis traps and replication crisis.. Eur. J. Nucl. Med. Mol. Imaging.

[99] Galavis P.E., Hollensen C., Jallow N., Paliwal B., Jeraj R. (2010). Variability of textural features in FDG PET images due to different acquisition modes and reconstruction parameters.. Acta Oncol..

[100] van Timmeren J.E., Leijenaar R.T.H., van Elmpt W., Reymen B., Lambin P. (2017). Feature selection methodology for longitudinal cone-beam CT radiomics.. Acta Oncol..

[101] Baeßler B., Weiss K., Pinto dos Santos D. (2019). Robustness and reproducibility of radiomics in magnetic resonance imaging.. Invest. Radiol..

[102] Zwanenburg A., Abdalah M.A., Apte A., Ashrafinia S., Beukinga J., Bogowicz M., Dinh C.V., Götz M., Hatt M., Leijenaar R.T.H., Lenkowicz J., Morin O., Rao A.U.K., Socarras Fernandez J., Vallières M., Van Dijk L.V., Van Griethuysen J., Van Velden F.H.P., Whybra P., Troost E.G.C., Richter C., Löck S. (2018). PO-0981: Results from the image biomarker standardisation initiative.. Radiother. Oncol..

[103] Rudin C. (2019). Stop explaining black box machine learning models for high stakes decisions and use interpretable models instead.. Nat. Mach. Intell..

[104] Ghassemi M., Oakden-Rayner L., Beam A.L. (2021). The false hope of current approaches to explainable artificial in health care.. Lancet Digit. Health.

[105] Reddy S. (2022). Explainability and artificial intelligence in medicine.. Lancet Digit. Health.

[106] Kujur A., Raza Z., Khan A.A., Wechtaisong C. (2022). Data complexity based evaluation of the model dependence of brain MRI images for classification of brain Tumor and Alzheimer’s disease.. IEEE Access.

[107] Khan A.A., Mahendran R.K., Perumal K., Faheem M. (2024). Dual-3DM3AD: Mixed transformer based semantic segmentation and triplet pre-processing for early multi-class Alzheimer's diagnosis.. IEEE Trans Neural Syst Rehabil Eng.

[108] Rastogi D., Johri P., Donelli M., Kadry S., Khan AA., Espa G., Feraco P., Kim J. (2025). Deep learning-integrated MRI brain tumor analysis: Feature extraction, segmentation, and survival prediction using replicator and volumetric networks.. Sci Rep.

[109] Khan A.A., Madendran R.K., Thirunavukkarasu U., Faheem M. (2023). D2PAM: Epileptic seizures prediction using adversarial deep dual patch attention mechanism.. CAAI Trans. Intell. Technol..

